# Chronic Pancreatitis Complicated by Pseudoaneurysm Causing Gastrointestinal Bleeding

**DOI:** 10.7759/cureus.85058

**Published:** 2025-05-29

**Authors:** Brandon Y Boeur, Hussein Haidari, Sagar Kumar

**Affiliations:** 1 Internal Medicine, University of South Alabama, Mobile, USA; 2 Pulmonary Critical Care, University of South Alabama, University Hospital, Mobile, USA

**Keywords:** bleeding, celiac trunk, embolization, pancreatitis, pseudoaneurysm

## Abstract

Patients with chronic pancreatitis frequently present with acute flares, which can lead to a variety of complications due to the underlying inflammation. Chronic pancreatitis leads to an inflammatory cascade, possibly leading to pseudoaneurysm formation. Due to its location, the branches of the celiac trunk are the most commonly affected arteries. The pseudoaneurysm can lead to a gastrointestinal bleed. Identification of these pseudoaneurysms early on in the clinical course is crucial to providing the appropriate management, as not all medical centers may be equipped with capabilities for artery coil embolization. In the present case, a female in her 50s presented for an acute flare of her chronic pancreatitis. During her hospitalization, she had an acute drop in her hemoglobin, prompting upper endoscopy by gastroenterology services to locate the source. Blood was visualized in the duodenum but without a clear source. A computed tomography angiography was performed, and a left gastric artery pseudoaneurysm was noted. The patient underwent coil embolization of the left gastric pseudoaneurysm. The patient’s hemoglobin levels stabilized post-procedure and was able to be discharged soon after the procedure.

## Introduction

Pancreatitis is an inflammatory condition of the pancreas that can present in both acute and chronic forms. Chronic pancreatitis, often linked to alcohol use disorder, can lead to a variety of complications, including vascular complications, such as pseudoaneurysms [1]. Other causes of chronic pancreatitis include genetic causes as well as repeated episodes of acute pancreatitis. The inflammatory process can weaken adjacent blood vessel endothelium, resulting in their erosion and the formation of pseudoaneurysms, which may rupture and lead to gastrointestinal bleeding. Such vascular complications are significant, as they can present atypically, complicating the diagnosis and management of the underlying pancreatitis. Up to 200,000 cases of pancreatitis are admitted in the United States of America every year. A quarter of patients with pancreatitis can develop vascular complications [2].

The affected arteries are typically in close proximity to the pancreas. The splenic artery (30-50%) is most affected, followed by the left gastric, gastroduodenal artery, and pancreaticoduodenal artery. The superior mesenteric, proper hepatic artery, and small intrapancreatic arteries may also be affected but are not as common. Early diagnosis of pseudoaneurysm is important to avoid life-threatening hemorrhage. Clinicians should be aware of pseudoaneurysm as a potential complication of pancreatitis. Diagnosis based on clinical presentation can be challenging given the overlap of symptoms, with hematemesis and abdominal pain being present in both conditions. Advanced imaging modalities, including computed tomography (CT) and computed tomography angiography (CTA), play a crucial role in identifying vascular complications and informing the management plan [3,4].

This report discusses the presentation, diagnostic approach, and management of a patient with chronic pancreatitis who developed a left gastric artery pseudoaneurysm leading to gastrointestinal bleeding, emphasizing the critical need for timely intervention in such cases.

## Case presentation

Over the course of two days, a 50-year-old woman with a history of chronic pancreatitis and alcohol use disorder came with intense core abdominal pain that spread to her back, consistent with her prior pancreatitis episodes. Her complete blood count (CBC) and comprehensive metabolic panel (CMP) were both normal at admission, and her vital signs were stable. Her lipase was noted to be elevated on admission, and she was treated for acute-on-chronic pancreatitis with intravenous fluid resuscitation, analgesic medication, and antiemetics. 

Her hospital course was complicated on the fifth day of hospitalization, as she experienced melena and had an acute hemoglobin level drop from 12 mg/dL to 10 mg/dL. She did not develop any hypotension or tachycardia. She was started on IV proton pump inhibitors, and two 18-gauge peripheral IVs were placed. Gastroenterology services were consulted for further evaluation, and the patient underwent an upper GI endoscopy. While the patient remained hemodynamically stable, upper GI endoscopy was pursued due to the large volume of melena. Frank blood was noted during the procedure, with no clear source of bleeding.

**Figure 1:**
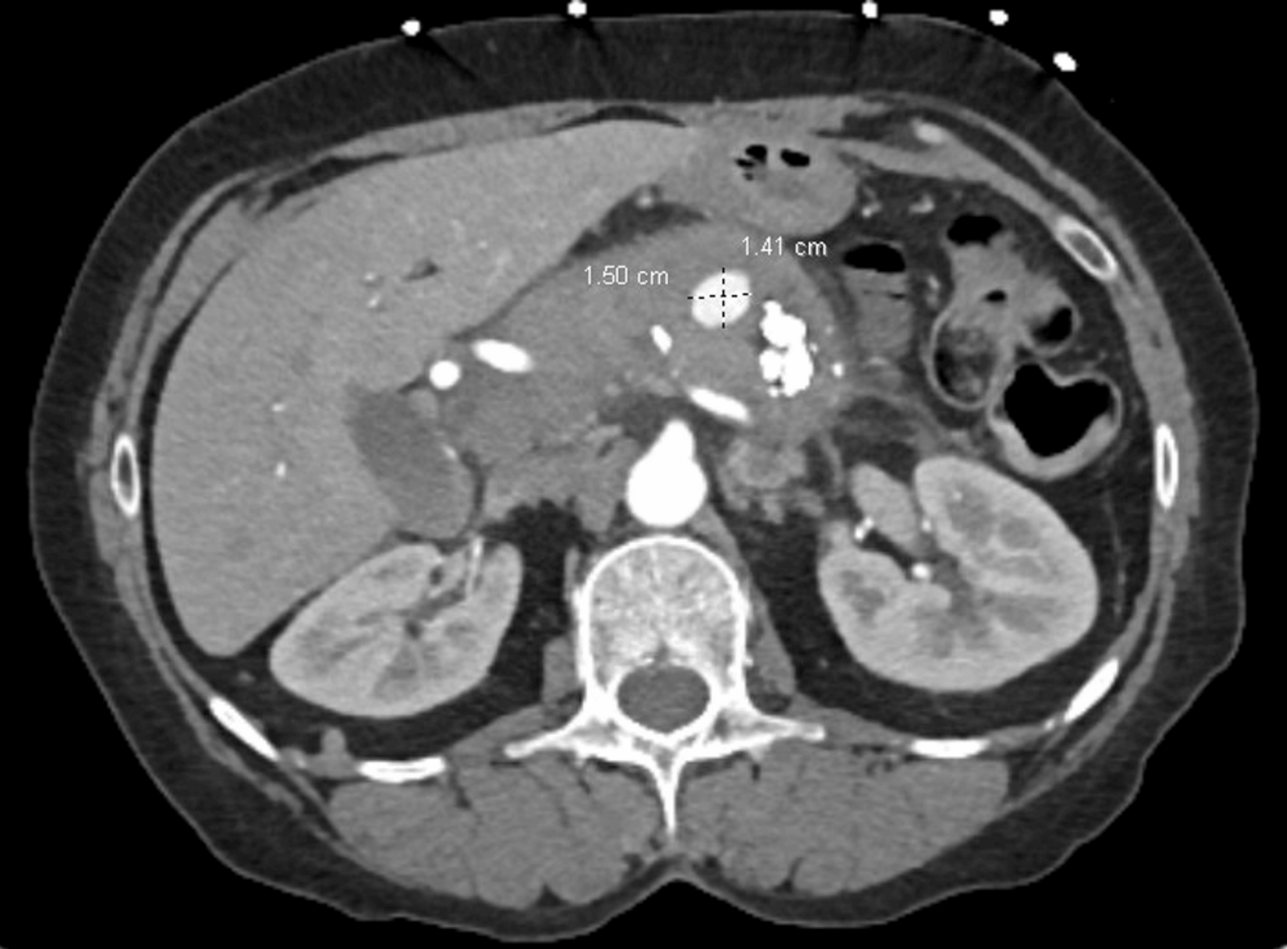
Left gastric artery pseudoaneurysm. CT angiogram of the abdomen and pelvis with intravenous contrast (axial view). Noted to be measuring 1.5 × 1.4 cm.

Due to blood loss of unknown origin, a CT-angiogram of the abdomen and pelvis was performed. A left gastric artery pseudoaneurysm, sized roughly 1.5 × 1.4 cm, was discovered by subsequent computed tomography angiography. There was no indication of contrast blush. Additional findings of a collection of pancreatic multicystic heterogeneous fluid were also observed (Figure 1).

**Figure 2:**
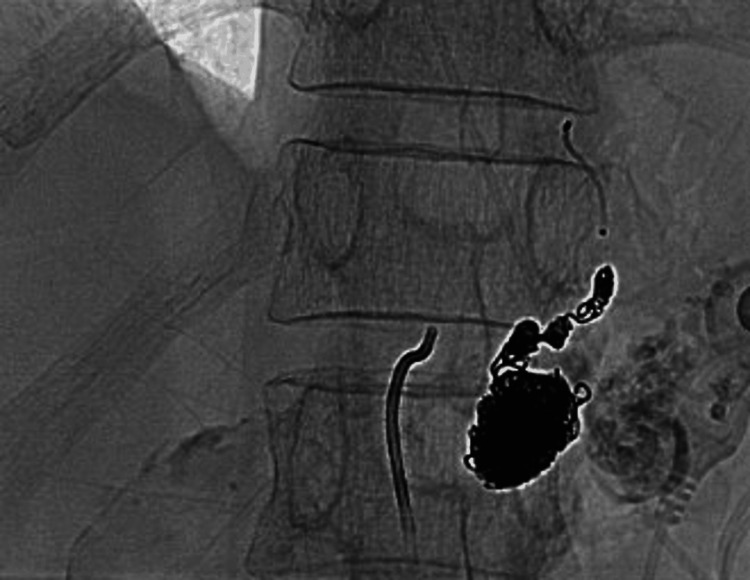
Coil embolization of left gastric artery. Packing coils noted of the left gastric artery.

Interventional radiology services were consulted at the time of discovery of the pseudoaneurysm. A 6-French vascular sheath was then introduced into the right common femoral artery. The celiac arterial system was then visualized with a 5-French Mickelson catheter (Cook Medical Inc.: Bloomington, IN). The pseudoaneurysm was then coiled with 4 mm × 60 cm packing coils and two ruby 3 mm × 20 cm coils. The patient's left gastric artery pseudoaneurysm was successfully and painlessly coil embolized (Figure 2).

Post embolization success confirmed with no noted contrast flow through the system for at least five heartbeats. Her hemoglobin levels were stable after the procedure, and her clinical condition significantly improved. She was able to be discharged with close primary care follow-up. This case emphasizes how crucial it is to detect and treat chronic pancreatitis as soon as possible, especially when vascular anomalies like pseudoaneurysms are present.

## Discussion

Pancreatitis-associated pseudoaneurysms (PPAs) are a rare consequence of chronic pancreatitis caused by repeated inflammation, which causes degradation of nearby blood vessels. Patients with a history of persistent alcohol use are potentially more prone to this complication as they are more prone to recurrent episodes of acute pancreatitis. In this study, a patient with chronic pancreatitis presented initially with concerns for acute-on-chronic pancreatitis. However, during hospitalization, the patient presented with gastrointestinal bleeding after developing a pseudoaneurysm in the left gastric artery. Given the subtlety of symptoms in pseudoaneurysm formation, this case highlights the significance of early identification and action to prevent fatal hemorrhagic episodes.

As seen in this instance, pseudoaneurysms in pancreatitis can impact the splenic, gastroduodenal, pancreaticoduodenal, and left gastric arteries [2,4]. The most common artery affected is the splenic artery (30-50%) [5]. PPAs can be difficult to diagnose because of their vague and subtle clinical manifestations. Early identification is difficult since patients often present with symptoms that are also typical in pancreatitis itself, such as abdominal pain, gastrointestinal bleeding, or, in extreme cases, hemodynamic instability [6].

Pathophysiology and risk factors

The pathophysiology of pseudoaneurysm development in chronic pancreatitis relates to the recurrent inflammation. Recurrent inflammation is a hallmark of chronic pancreatitis, and pancreatic enzymes like trypsin and elastase leak out of the pancreatic ducts to break down surrounding tissues [7]. This causes arterial wall erosion over time, which puts vessels at risk for developing pseudoaneurysms. Chronic alcohol consumption, recurrent bouts of pancreatitis, and prior surgical or interventional procedures are significant risk factors for pseudoaneurysm formation [8]. Because persistent alcohol misuse is strongly linked to chronic pancreatitis and its associated vascular complications, it is considered a significant risk factor [9].

Clinical presentation and diagnostic approach

The location of the pseudoaneurysm, size, and extent of vessel wall erosion can all have a substantial impact on how PPA presents. The patient's significant abdominal discomfort was followed by melena and a drop in hemoglobin, which indicated underlying gastrointestinal bleeding. Although gastrointestinal bleeding is a typical sign of PPA, it is sometimes vague, making early detection more difficult [10]. This case serves as an example of the diagnostic challenge; although endoscopy showed duodenal blood, the source of the bleeding was not identified until further imaging revealed a pseudoaneurysm in the left gastric artery.

Due to their ability to provide a thorough picture of both pancreatic inflammation and vascular abnormalities, computed tomography (CT) and computed tomography angiography (CTA) play a critical role in the diagnosis of PPA, with CTA being the more preferred imaging modality [11]. High sensitivity PPA detection, vascular delineation, and interventional planning are all made possible by CT and CTA. In this instance, CTA guided subsequent embolization by detecting a 1.5 × 1.4 cm pseudoaneurysm in the left gastric artery. Although CTA is still the recommended method, other modalities, including Doppler ultrasound and magnetic resonance angiography, may be helpful in specific circumstances [12,13]. CTA is an important modality to consider in patients who develop clinical instability and may not tolerate upper GI endoscopy. In addition, if an upper GI endoscopy does not reveal a clear source of bleeding, CTA can help elucidate the etiology.

Management and interventional approach

Management of PPA primarily involves endovascular methods, with coil embolization being the preferred treatment due to its high success rates and minimally invasive nature. In this instance, the patient successfully underwent coil embolization of the pseudoaneurysm located in the left gastric artery, aligning with established best practices [14]. This procedure effectively occludes the pseudoaneurysm, stopping any bleeding and preserving blood flow to the surrounding tissue, making it a more favorable choice than surgical alternatives. When endovascular methods are ineffective or not an option, open surgical interventions such as vessel ligation or resection might be considered, though these approaches come with increased risks and longer recovery times [15].

The effectiveness of endovascular treatment for PPAs is notable, with research indicating a hemostasis success rate of 80-90% via coil embolization [16]. Potential complications can occur, such as ischemic damage to nearby tissues, infections, and in certain cases, the pseudoaneurysm may return. For this patient, stable hemoglobin levels after the procedure indicated that the intervention was successful and free of significant complications. Continued monitoring is advised for these patients, as there is a risk of pseudoaneurysm recurrence due to persistent inflammation, especially if the patient continues to drink alcohol [17]. However, this may not be available in areas without the resources to accommodate surveillance with repeat CTA imaging.

Prevention and long-term outlook

Patients with chronic pancreatitis must have their risk factors changed and the underlying condition aggressively managed in order to prevent PPA. Reducing the chance of recurrence requires addressing persistent alcohol use, which is a known risk factor for chronic pancreatitis. In order to manage chronic pancreatitis and avoid its consequences, pharmacologic therapies, such as pancreatic enzyme replacement therapy, dietary adjustments, and lifestyle changes, are crucial [18]. A key component of this preventive strategy is alcohol abstinence and alcohol dependency treatment, which may lessen the pancreatic inflammatory load.

In order to manage pancreatitis, lower the risk of recurrence, and treat alcohol dependence, this patient would benefit from a multidisciplinary strategy that includes gastroenterologists, interventional radiologists, and potentially addiction specialists. Patients at high risk of pseudoaneurysm recurrence may also benefit from periodic imaging surveillance, usually with CT or ultrasound, to enable early management in the event of new vascular anomalies [19,20].

## Conclusions

Even though pseudoaneurysms associated with pancreatitis are uncommon, they are serious complications of chronic pancreatitis. This case highlights the importance of early diagnosis and appropriate treatment. The importance of advanced vascular imaging protocols such as CTA and endovascular procedures is demonstrated in this case by the successful diagnosis and coil embolization of a pseudoaneurysm in the left gastric artery. It must be noted that not all cases would benefit from endovascular procedures, as the anatomy can vary. This case also emphasizes the necessity of a preventive strategy to address chronic alcohol consumption, a significant modifiable risk factor. Clinicians should consider a multidisciplinary approach to increase the effectiveness of risk factor reduction by potentially involving an addiction specialist. When patients with chronic pancreatitis present with unexpected hemoglobin reductions or gastrointestinal bleeding, clinicians should maintain a high index of suspicion for pseudoaneurysms.
